# Effectiveness of Treatment of Periapical Lesions in Mature and Immature Permanent Teeth Depending on the Treatment Method Used: A Critical Narrative Review Guided by Systematic Principles

**DOI:** 10.3390/jcm14145083

**Published:** 2025-07-17

**Authors:** Aleksandra Jankowska, Wojciech Frąckiewicz, Agnieszka Kus-Bartoszek, Aleksandra Wdowiak-Szymanik, Anna Jarząbek

**Affiliations:** 1 PeriDent—Dentistry and Periodontology Center, Przecław 119A, 72-005 Przecław, Poland; 2 Apolonia Dental, Gryfińska Street 62A, 70-772 Szczecin, Poland; 3 Independent Pediatric Dentistry Clinic, Powstańców Wielkopolskich Avenue 72, 70-111 Szczecin, Poland

**Keywords:** treatment effectiveness, endodontic treatment, apexification, revitalization, periapical lesions, MTA, Ca(OH)_2_

## Abstract

This critical narrative review presents the concepts and methods that have been or are currently applied in the treatment of periapical tissue changes in mature and immature permanent teeth. Treatment success is defined as the healing of the inflammatory lesion in permanent teeth and, additionally, the completion of root development in immature teeth. **Background/Objectives:** Endodontics focuses on the prevention and treatment of diseases affecting the dental pulp and periapical tissues. Periapical changes have been managed using various methods depending on factors such as the extent of the lesion and the stage of root and apical development. Conventional root canal treatment, revitalization, and apexification have all been employed. **Methods:** Three databases (PubMed, Scopus, and Web of Science) were searched for studies discussing different treatment approaches, materials, and the efficacy of techniques used over time in mature and immature permanent teeth. **Results:** This review includes seven case reports, seven case series, and three cohort studies, each detailing the treatment method, case characteristics, follow-up period, and treatment outcomes. **Conclusions:** Modern materials have significantly improved the outcomes of revitalization and apexification procedures. These methods can now compete with or even surpass the clinical effectiveness of conventional root canal therapy.

## 1. Introduction

Endodontics is a branch of dentistry focused on preventing and treating diseases of the endodontium—a complex structure consisting of the pulp and dentin—as well as the apical tissues of the tooth. The dental pulp and periapical tissues are closely related anatomically, topographically, and functionally. Pulp diseases are most commonly caused by bacterial infections. Bacteria can enter the endodontium through various pathways, including carious lesions or traumatic injuries [1,2].

### 1.1. Formation of Periapical Lesions

**Figure 1:**
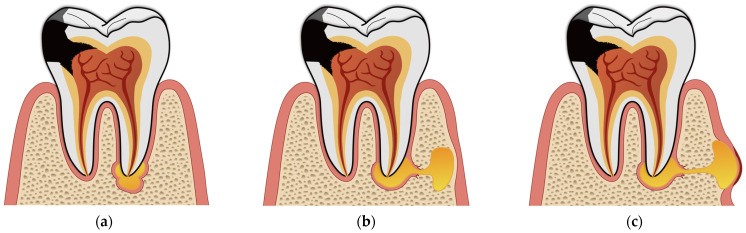
Types of odontogenic abscesses: (**a**) periapical; (**b**) subperiosteal; (**c**) submucosal.

Periapical lesions are inflammatory conditions affecting the bone and periodontal tissues surrounding the apices of the teeth. In the acute stage, they typically present as toothache [3], accompanied by a periapical, subperiosteal, or submucosal abscess, depending on the progression of the lesion (Figure 1). Additional classic signs of inflammation—such as redness, swelling, and an elevated temperature of the gingiva and surrounding tissues—may also be present. In cases of chronic inflammation, pain is usually reduced, as the inflammatory process creates a drainage pathway from the pulp chamber to the periapical tissues. However, the lesion may enlarge and spread to adjacent areas. An intraoral fistula—or, less commonly, an extraoral one—can also develop, allowing the drainage of purulent material [4]. Radiographically, the lesion typically appears as the localized thinning of the bone structure. In more advanced cases, spherical radiolucencies may form, indicating the presence of granulomas or cysts [5].

Primary inflammation of the periapical tissues can occur in any tooth and may affect one, several, or all canals of a given tooth [6]. The most common causes include dental caries or trauma. In cases of tooth fracture, if the inflammation does not resolve spontaneously, endodontic intervention becomes necessary [7]. Secondary inflammation affects teeth that have previously undergone root canal treatment. It may result from retained necrotic, bacterially infected pulp tissue; inadequate canal preparation or obturation; anatomical variations; undetected canals; or other procedural errors during endodontic therapy [8,9].

The diagnosis of periapical inflammation begins with identifying the affected tooth. Patients usually have no difficulty determining the side of the pain, but distinguishing whether it originates from the maxillary or mandibular arch can be more challenging. Pain from maxillary teeth typically radiates to the temples and eyes, while pain from mandibular teeth may radiate to the neck, occipital region, or nape [4]. Further diagnostic procedures involve pulp vitality tests—such as thermal and electric pulp testing—as well as periapical tissue assessments, including palpation and percussion tests [10,11].

Radiological examinations are a standard component in diagnosing pulp diseases. Depending on the case, a panoramic radiograph may be taken initially, followed by a targeted periapical radiograph [11]. In more severe cases, cone beam computed tomography (CBCT) is used to image the affected area. These imaging techniques help to determine the stage of the lesion, such as chronic periapical inflammation, granuloma, or a cyst [12,13].

Immature permanent teeth are still in the process of development. They have less mineralized dentin, wider dentinal tubule lumens, pulp horns positioned closer to the enamel–dentin junction, and incompletely formed roots and apices. These characteristics make the pulp more vulnerable to bacterial invasion and increase the teeth’s sensitivity to destructive and irritating factors.

### 1.2. Evolution of Treatment Methods

The history of treating periapical lesions in immature permanent teeth dates back to the mid-20th century. The classical apexification technique involved the induction of an apical barrier through multiple applications of calcium hydroxide. Although effective, this method was time-consuming and required numerous clinical visits [14]. In the 1990s, mineral trioxide aggregate (MTA) was introduced, which addressed the issue of prolonged treatment by enabling apical closure in a single visit. Additionally, MTA demonstrated superior sealing abilities and biocompatibility compared to calcium hydroxide [15]. A further advancement in the materials used for apexification came with the development of modern bioceramics such as Biodentine, which offer high bioactivity, easier handling, and faster setting times compared to MTA [16]. Regenerative endodontic procedures, particularly revascularization, represent the next step in therapeutic evolution. This biologically based approach combines advanced bioceramic materials with tissue regeneration, moving beyond mere mechanical apical barrier formation. Revascularization is a sophisticated regenerative method that utilizes stem cell recruitment and promotes the healing of periapical tissues along with continued apical development [17].

### 1.3. Currently Used Treatment Methods

The treatment of periapical inflammation in mature permanent teeth typically involves conventional root canal therapy. This procedure includes removing the pulp from the tooth chamber, followed by chemo-mechanical preparation using files and irrigation solutions and, finally, sealing the canal tightly with a filling material.

In the treatment of periapical inflammation in immature permanent teeth, the apexification method is commonly used [18]. This approach involves materials such as calcium hydroxide paste, which helps to dry and neutralize the acidic pH of inflamed tissues [19], or MTA or bioceramic materials like Biodentine [20]. According to the European Society of Endodontology (ESE) [21], apexification with calcium hydroxide promotes continued root development and often leads to complete root formation or at least the creation of an apical barrier. However, the prolonged treatment time and the frequent need to replace the canal dressing often result in root wall weakening and fractures, leading to treatment failure. Currently, apexification with MTA is the preferred method. This procedure involves the careful chemo-mechanical preparation of the canals, followed by clinical assessment. In the absence of pain, a 3–5-mm-thick layer of MTA is placed around the canal apex. Condensation is performed using appropriate pluggers, paper points, and spreaders. After radiographic verification, the clinician temporarily seals the canal with a moist cotton pellet to allow the MTA to set. The remaining canal space is typically filled using the warm vertical compaction of the gutta-percha technique, and the coronal defect is restored with composite resin or prosthetic reconstruction.

One of the newer materials, Biodentine is a calcium silicate-based material developed to retain the beneficial properties and therapeutic applications of MTA while minimizing the associated risks. Its improved clinical handling offers safer use, eliminates the need for a two-step obturation process, accelerates the setting time, and reduces the risk of bacterial contamination. For these reasons, Biodentine is considered superior to MTA. This versatile material can be used for root perforation repair, the treatment of internal and external resorption, apical barrier formation during apexification, regenerative procedures, pulp capping, pulpotomy, dentin replacement in coronal restorations, and as a retrofilling material in endodontic surgery [22,23,24].

Another relatively recent, albeit less commonly used, alternative to apexification is revitalization, also known as pulp regeneration [25]. This method employs a three-antibiotic paste (TAP, consisting of metronidazole, ciprofloxacin, and minocycline), a two-antibiotic paste (DAP, which excludes minocycline) [26], or, more recently, calcium hydroxide. These materials enable the thorough disinfection of the root canals. Through subsequent stages of revitalization, pulp-like tissue forms within the canal, aiming to restore blood circulation in the pulp chamber and promote continued root development. Although some clinicians have attempted to apply revitalization in mature permanent teeth, conventional root canal treatment remains the most established method in such cases. Originally termed revascularization, this name referred specifically to the restoration of proper blood flow or the revascularization of tissue damaged by pathological processes such as trauma or caries. However, the terminology related to the tissue formed after pulp regeneration has been debated. For instance, Huang et al. [27] argued that the term does not fully describe all the changes occurring during the process. Following revitalization, the tooth may regain the ability to respond to pain stimuli due to the partial regeneration of nervous tissue. Additionally, structures such as cementum, dentin, or bone-like tissue may form, with cells migrating from the periapical area, contributing to the regeneration process. Revitalization can result in an increased root length and thickness, a process known as maturogenesis, which ultimately improves the root strength.

### 1.4. Mechanisms of Tissue Regeneration

A detailed understanding of the molecular and biological mechanisms of materials used in the treatment of teeth with immature apices enables the more precise development of clinical protocols. Mineral trioxide aggregate (MTA) exhibits strong biological activity—during setting, it releases calcium ions and maintains a high pH, which initiates the formation of a calcium silicate hydrate gel. In vitro studies have shown that MTA significantly upregulates the expression of genes such as Runx2 and osterix in mesenchymal stem cells, thereby promoting osteogenic and chondrogenic differentiation and facilitating the formation of a hard-tissue apical barrier—an essential stage in the process of apexification [28].

Antibiotic pastes, on the other hand, play a crucial role in eliminating pathogens from the root canal, which is necessary to establish a sterile environment conducive to tissue regeneration [29]. However, their effects on stem cells are highly concentration-dependent: high concentrations (e.g., 1000 mg/mL) have been shown to be cytotoxic, reducing cells’ viability, proliferation, and differentiation capacity [30], whereas lower concentrations may support the function of stem cells from the apical papilla (SCAP) if properly adjusted.

Calcium hydroxide (Ca(OH)_2_), traditionally used in apexification, remains a biocompatible material. Its high pH supports SCAP survival and differentiation, while also initiating mineralization. Studies have shown that Ca(OH)_2_ is non-toxic at clinically relevant concentrations, making it a safe and effective agent in regenerative procedures such as apexification and revascularization [31]. Understanding these mechanisms allows for the more biologically guided and evidence-based customization of therapeutic strategies in the management of immature teeth with necrotic pulps.

## 2. Materials and Methods

This review was designed to include case reports and case series, which provide detailed descriptions of individual procedures and cohort studies that evaluate the effectiveness of different treatment methods in comparison to one another. The authors aimed to select articles not only from the recent literature but also from earlier years, in order to analyze how the approaches and materials have evolved over time.

### 2.1. Literature Search Strategy

To identify relevant studies published between January 1980 and May 2025, a non-systematic search was conducted through three databases: PubMed, Scopus, and Web of Science. The search strategy combined Medical Subject Headings (MeSH) terms and text words related to the treatment of periapical lesions in permanent mature and immature teeth, as well as associated treatment options. The MeSH terms included “Apexification”, “Revascularization”, “Conventional Root Canal Treatment”, “Immature Permanent Teeth”, “Permanent Teeth”, and “Periapical Lesions”. Eligible studies for screening included case reports, case series, and cohort studies, limited to publications in English. The full search strategy is provided in the Supplementary Materials (Table S1).

### 2.2. Inclusion and Exclusion Criteria

Inclusion criteria:

- Studies evaluating treatment outcomes of periapical lesions in mature or immature permanent teeth;
- Human studies;
- Articles reporting on clinical and/or radiographic outcomes;
- Studies comparing different treatment methods (e.g., regenerative endodontics, apexification, conventional root canal therapy).

Exclusion criteria:

- Animal or in vitro studies;
- Editorials, cover letters to the editor, and abstracts without full texts;
- Studies with insufficient outcome data.

**Figure 2:**
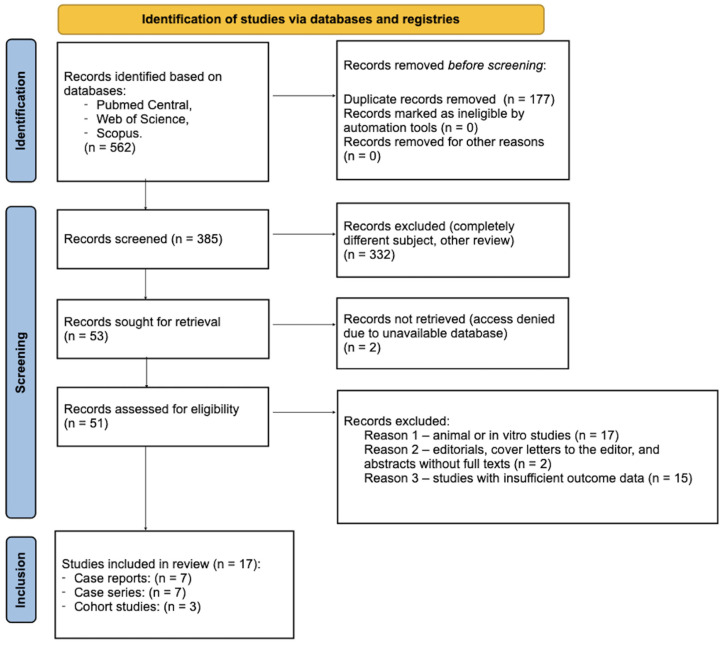
PRISMA flow chart of study selection.

After removing duplicates, the titles and abstracts of the identified studies were screened independently by two reviewers (A.J. and A.K.-B.). Potentially eligible articles were assessed in full text for inclusion. Any disagreements were resolved through discussion. The PRISMA 2020 [32] flow diagram below (Figure 2) illustrates the number of studies identified, screened, assessed for eligibility, and included in the review, along with reasons for exclusion at each stage.

### 2.3. Data Extraction

From each included article, the following data were extracted:

- Author(s), year of publication;
- Study design and number of cases;
- Type of tooth (mature/immature);
- Treatment method used;
- Follow-up duration;
- Clinical and radiographic outcomes;
- Reported success or failure rates.

The narrative synthesis of the research was carried out following a thorough screening procedure and data extraction. 

### 2.4. Quality Assessment

Although this review was narrative in nature, we included an assessment of the methodological quality for transparency. To assess the methodological quality of the included studies, separate tools were used according to the study design.

To assess the methodological quality of the included studies, we employed the JBI critical appraisal checklists. The JBI checklist for case reports [33] was used for single case studies (Table 1), while the checklist for case series [34] was applied to studies involving multiple cases (Table 2). For cohort studies, the JBI critical appraisal tool for cohort studies [35] was utilized (Table 3). Each study was independently assessed based on a series of questions addressing potential sources of bias. The quality assessment was conducted based on responses to the JBI critical appraisal checklist, with answers categorized as “Yes”—indicating that the criterion was met, “No”—indicating that the criterion was not met, and “N/A or partially”—indicating insufficient information to make a judgment. The proportion of “Yes” responses relative to the total number of items was calculated to yield a percentage score. Based on this score, the methodological quality was classified as high (≥80%), moderate (60–79%), or low (<60%). This classification provides a concise and standardized measure of the reliability and methodological rigor of the included studies. The quality assessment results were summarized in a tabular format, allowing for comparison across studies and the identification of the most common methodological limitations (Table 4).

Studies were independently assessed by two reviewers (A. Jankowska and W. F.), and discrepancies were resolved through discussion or consultation with a third reviewer (A. Jankowska) when necessary.

## 3. Results

This review includes seven case reports [36,37,38,39,40,41,42] and seven case series [43,44,45,46,47,48,49] that provide detailed descriptions of the treatment of large periapical lesions in permanent teeth, including the procedures used and follow-up outcomes. Additionally, this article discusses three cohort studies [50,51,52] that compare different treatment methods for periapical lesions within each study.

The treatment options for large periapical lesions range from conventional non-surgical root canal therapy combined with long-term calcium hydroxide application to various surgical interventions. In the reviewed articles, large periapical lesions in permanent teeth were treated using conventional endodontic therapy, the Endo-eze system, apexification, and revitalization methods.

Table 5 presents the articles categorized by type of study: case reports, case series, and cohort studies. 

### 3.1. Revitalization Method

**Figure 3:**
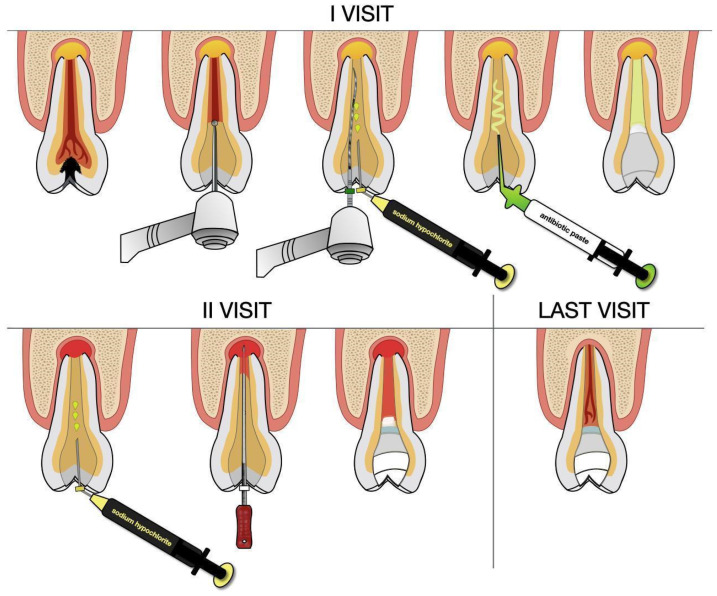
An indicative scheme of revitalization using antibiotic paste and MTA material.

Regenerative endodontic procedures (REP) were initially proposed for the treatment of immature permanent teeth, as they promote further root development, increase the root wall thickness, and facilitate apical closure. Currently, the application of REP has been extended to mature teeth because, if pulp-like tissue with functions similar to living pulp is restored, the tooth regains its resistance and there is a possibility to limit infections within the root canal. In contrast, teeth treated with conventional root canal therapy (CRCT) lose this function, as the pulp is replaced with root canal filling materials. For REP to be successful, stem cells from the apical area must migrate into the canal. An example of a revitalization protocol using antibiotic paste and MTA is illustrated in Figure 3. In cases of successful treatment, radiographic evidence of healing can be observed within 6 to 12 months [53]. This method was applied to mature permanent teeth by Arslan et al. [51], Paryani and Kim [43], Saoud [44], and Nageh [45]. Paryani and Kim [26], Saoud [44], and Nageh [45] reported the complete healing of inflammatory lesions in the apical region in all examined teeth after 12 to 22 months, while Arslan et al. [51] achieved favorable treatment outcomes in 92.3% of cases. The studies included in this review demonstrate that pain symptoms completely resolved following this treatment.

After the procedure, the canal develops pulp-like tissue, performing functions similar to those of living pulp. The criteria for assessing the effectiveness of this procedure are clearly defined by the European Society of Endodontology (ESE) [21] and include the healing of inflammatory changes, the absence of clinical symptoms, and a positive pulp response in sensitivity tests. As shown in this review, the healing of inflammatory changes and a lack of clinical symptoms were achieved, and a positive pulp response to cold and electric tests was observed in 30 teeth included in our analysis. According to the American Association of Endodontists (AAE) guidelines, the primary goals of revitalization are healing inflammation in the apical periodontal region and the elimination of clinical symptoms—both of which were met in the cited studies. Other goals, such as continued root development in immature permanent teeth and positive pulp sensitivity tests, are desirable but not essential in determining treatment success [54]. The revitalization procedures described by the authors followed the recommended protocols. During the first visit, after chemo-mechanical preparation, the canals were temporarily filled with various materials: a triple-antibiotic paste (consisting of ciprofloxacin, metronidazole, and doxycycline) [51], ciprofloxacin alone [43], or a dual-antibiotic paste (ciprofloxacin and metronidazole) [45]. Paryani and Kim [43] used calcium hydroxide for canal disinfection, while Saoud [44] used a metapaste composed of Ca(OH)_2_ and barium sulfate.

In one of the systematic reviews [55], in which the authors compared the effects of all substances used for revascularization, a high success rate in healing periapical lesions was demonstrated for all methods used. Vatankhak et al. [26] compared the effects of antibiotic pastes and found that single-antibiotic pastes (SAP) were characterized by significantly higher or similar antibacterial efficacy compared to double (DAP), triple (TAP), or calcium hydroxide. This allowed them to conclude that combined antibiotic therapy is not necessary for ex vivo root canal disinfection. In the presented studies, the efficacy of intracanal inserts was high. The choice of Ca(OH)_2_ is supported by the lack of antibiotic resistance and the lack of allergic reactions [21,37]. The results of other studies support the choice of a combined triple-antibiotic paste, as it proved to be more effective in treating the most resistant root canal infections, and the healing of inflammatory lesions in the apical area after its use was faster compared to calcium hydroxide [56].

The authors used NaOCl in the following concentrations for canal irrigation: 5.25% [43], 2.5% [44], 1.5% [45], and 1% [51]. The literature data indicate that it can be used in this procedure in a concentration of 0.5–5.25%. The AAE recommends a 1.5% solution, because higher concentrations of sodium hypochlorite could damage stem cells, which play an important role in tissue regeneration. Low concentrations of sodium hypochlorite should be compensated for by an increased volume of the solution (20 mL in 5 min).

The criteria for the success of revitalization according to the ESE also include a lack of tooth discoloration and the patient’s acceptance of the effect [21]. The development of crown discoloration after revitalization is associated, among other aspects, with the use of minocycline in a triple-antibiotic paste, which is why Thibodeau et al. [57] and Dabbagh et al. [58] recommend replacing minocycline with cefaclor. Clindamycin or amoxicillin can also be used instead of minocycline. In the presented cases, minocycline was used only by Arslan et al. [51], but, in order to avoid discoloration, the authors covered one third of the canal interior with resin (Futurabond U, Voco, Cuxhaven, Germany) before introducing the triple-antibiotic paste.

The next stage of treatment is to induce bleeding in the canal by irritating the periapical tissues, beyond the root apex, with various types of files (25 K-file, 30 H-file, 40 K-file). This part of the procedure is performed under anesthesia. The ESE recommends the use of anesthetics without vasoconstrictors but, at the same time, notes that performing this part of the procedure is difficult when the patient is in pain. There is also little evidence in the literature that bleeding after the use of anesthetics without a vasoconstrictor is more effective. During the procedure, the experience of the patient’s compliance with the recommendations, their control of anxiety, and their pain during the first visit should be taken into account [21]. The authors mainly used mepivacaine without vasoconstrictors. Only Paryani and Kim [43], in two cases, anesthetized patients with 2% lidocaine with adrenaline. In the presented studies, the formed clot was covered with MTA material, which was typically placed about 2–4 mm below the CEJ [43] and had a thickness of about 3 mm [44]. Any hydraulic silicon cement can be used instead of MTA. To allow the material to harden, a wet cotton pad was placed on the MTA, which was then covered with a temporary material, and, at the next visit, after confirming that the plug had been formed correctly, it was closed with a final composite restoration. Paryani and Kim [43], in accordance with the ESE recommendations, covered the clot with a Collacote membrane before MTA placement, while Nageh et al. [45] covered it with a PRF membrane. It should be noted that the efficacy of the presented cases was comparable, and the additional use of membranes was not associated with higher treatment efficacy.

The length of treatment with revascularization involves at least two visits with an interval of 2–4 weeks. The time between visits is related to the disinfecting effect of the antibiotic paste or Ca(OH)_2_ in the canal. A greater number of visits may also be related to poor patient cooperation, as presented by Saoud et al. [44].

### 3.2. Conventional Endodontic Treatment

The most common method of treating periapical lesions is conventional endodontic treatment, which involves removing the inflamed or necrotic pulp and replacing it with root canal filling materials. Modern endodontics employs various canal preparation techniques, primarily categorized as crown-down and step-back methods [59], as well as different canal filling techniques, including the use of liquid gutta-percha or the single-cone method [60]. Conventional root canal treatment (CRCT) is the most widely used approach and has been performed on both permanent mature teeth [36,37,38,47,48,50,51] and immature teeth [50].

When discussing treatment success, it is important to distinguish between the following:

- Complete success, defined as the full healing of periapical lesions, the absence of pain, no crown discoloration, the disappearance of any fistulae, and complete apical closure around the infected tooth;
- Partial success, when one or more of the above criteria are not fully met.

**Figure 4:**
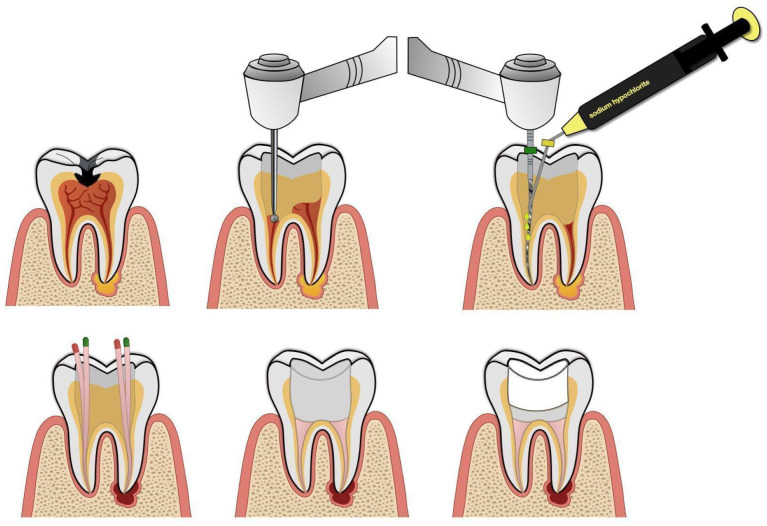
An indicative scheme of conventional root canal treatment.

Most of the studies included in our review that used conventional root canal treatment (CRCT) involved mature teeth. In these studies, the lesions generally healed successfully [36,37,47], except in the study by Ayna and Anya [38], where one tooth out of five treated in a single patient had to be extracted due to treatment failure. The time to complete healing ranged from 6 months to 2 years, although, in one study, lesion healing and reduction were observed as early as 3 months. On the other hand, Peretz and Yakir [39] reported less favorable results, with only 36% of cases achieving complete treatment success. They also noted that the success rates were significantly lower in younger patients. Ballikaya et al. [49] suggested that this might be because filling wider root canals takes more time, and the duration of the procedure heavily depends on patient cooperation. Additionally, treating immature teeth with wide apical openings carries a higher risk of irrigant extrusion and complicates canal filling. Arslan et al. [51] reported favorable clinical and radiographic outcomes in 92.3% of cases treated with regenerative endodontic procedures (REP)—the revitalization method described earlier—compared to 80% success for CRCT, with an overall success rate of about 73.4% at 12 months. Although this difference was not statistically significant, it suggests a potential advantage of regenerative treatment over conventional methods in immature teeth. An example diagram illustrating the individual steps of root canal treatment is shown in Figure 4.

Conventional root canal treatment (CRCT) involves removing the pulp from the tooth cavity, followed by chemo-mechanical preparation using files and canal irrigants and, finally, tightly filling the tooth cavity with a sealing material. Most authors in the reviewed studies used sodium hypochlorite (NaOCl) in concentrations ranging from 2.25% to 5.25% for canal irrigation [36,37,38,47,51]. Older studies reported the use of 2% chloramine, which is no longer commonly used [50]. Additionally, some studies used 3% hydrogen peroxide [50] to enhance canal disinfection, as well as 5% EDTA and distilled water [51]. According to the literature, in cases of heavily infected canals, chlorhexidine may be used, and EDTA can be replaced with about 40% citric acid. To improve treatment success and bacterial eradication, ultrasonic activation is currently recommended as it enhances the irrigation effectiveness [61]. Mechanical preparation in the studies included in our review was performed using both hand files [37,47,51] and rotary (machine) files [36,51]. Disinfection between visits is often necessary to relieve pain and reverse inflammatory changes in the bone. The treatment protocols reported ranged from two visits [37,50,51] to three visits [36] and multiple visits (6–7) [38,47], with intervals of about one week between appointments. Older studies used 2% chloramine for both irrigation and disinfection [50]. Calcium hydroxide was also used as a disinfectant [38,51], sometimes mixed with distilled water [37] or with glycerin in an 8:1 ratio [47]. Cheng and Liang [36] used Vitapex, a material composed of 30% calcium hydroxide, 41% iodoform, and 23% silicone oil.

The final canal filling was performed using gutta-percha with a lateral condensation technique, combined with various types of sealers—such as epoxy resins [37,38,51], calcium hydroxide [47], Klorpercha [50], or Cortisomol (gutta-percha with eugenol) [36]. This review included one study that covered both mature and immature teeth [50]. The authors applied the same methods, materials, and techniques for both groups. Among 40 incisors with partially closed apices and 33 with narrow apices treated with conventional root canal therapy, the overall success rate (without separating mature and immature teeth) was 60%. This success rate was significantly lower than that reported for apexification in the same study, which reached 95%.

### 3.3. Methods Using the Endo-Eze System

#### 3.3.1. Conventional Endodontic Treatment Using the Endo-Eze System

The treatment using the Endo-Eze system deserves particular attention. In the study by Meija et al. [46], case no. 2 involved a lesion exceeding 200 mm^2^. Under local anesthesia, an access cavity was prepared, and the Endo-Eze vacuum system was used to evacuate the exudate. The Endo-Eze vacuum is a high-volume suction aspirator connected to a microneedle. The negative pressure that it generates enables the evacuation of large amounts of inflammatory fluid from the periapical area. The root canals were cleaned, prepared, and irrigated with 5.25% sodium hypochlorite. The vacuum-assisted suction technique was repeated after 1, 2, and 4 weeks. The canine was filled with gutta-percha after one week, while the lateral incisor was obturated four weeks after the initial treatment. This approach led to a reduction in periapical changes and the resolution of symptoms within 3 months, with the complete healing of the lesion observed after 8 months.

#### 3.3.2. Apexification Method Using the Endo-Eze System

The Endo-Eze system can also be utilized in the treatment of immature teeth in combination with the apexification method. Meija et al. [46] applied this technique in clinical cases where a large amount of root canal exudate was present. The drainage procedure was repeated as needed during subsequent visits. The canal was irrigated similarly to that of mature teeth using 5.25% sodium hypochlorite, and the access cavity was sealed with a temporary restoration. After one week, calcium hydroxide was placed in the canal for a period of 8 to 14 months. Notably, no additional medication was introduced into the canals between these two visits. After 3 months, the authors observed a reduction in the lesion and evidence of continued root development. After 8 to 14 months (depending on the specific tooth), complete apexification and apical closure were achieved. Once root development was complete and the apex fully closed, the canals were obturated using thermoplasticized gutta-percha.

### 3.4. Apexification Method

**Figure 5:**
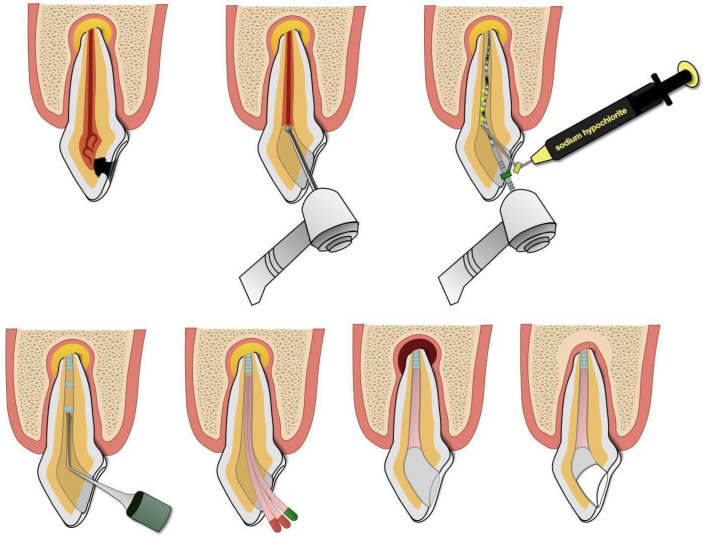
An indicative scheme of apexification using MTA material.

Permanent teeth with necrotic pulp and incomplete root development are typically treated using apexification with calcium hydroxide or mineral trioxide aggregate (MTA). In earlier approaches, products such as Metapex [39], which is based on calcium hydroxide and iodoform, were commonly used. More recently, calcium silicate-based materials like Biodentine [40,41] have been introduced for this type of procedure. According to the literature, calcium hydroxide can lead to successful apexification in approximately 95% of cases; however, it requires multiple visits, and the formation of a mineralized apical barrier can take anywhere from 3 to 24 months. A significant drawback of calcium hydroxide apexification is that the root walls often remain thin and brittle, which may compromise long-term tooth strength. This extended treatment duration poses particular challenges for younger patients. Scientific studies [62,63] have demonstrated that, during endodontic therapy, various bacterial species may infiltrate the root canal system from the oral cavity, potentially infecting the periapical tissues. Therefore, minimizing the number of visits is advised [64]. In a study by Cehreli et al. [42], two teeth underwent apexification using a protocol that involved irrigation with 2.5% sodium hypochlorite (NaOCl) and 2% chlorhexidine. Between appointments (four visits spaced weekly), dry, sterile cotton was placed in the pulp chamber. At the penultimate visit, the canal was sealed with a 4-mm-thick MTA plug at the apical end (1 mm short of the working length), and, at the final visit, the canal was obturated using thermoplasticized gutta-percha and AH Plus epoxy resin sealer. Although primarily intended for immature permanent teeth, one study [50] reported successful apexification in teeth with both open and closed apices. Mishra et al. [52] conducted a comparative study evaluating the use of ProRoot MTA with and without the addition of platelet-rich fibrin (PRF). Their results showed that the group treated with PRF-enhanced MTA experienced significantly faster healing and more rapid apical closure, suggesting that PRF may positively influence regenerative outcomes. A schematic representation of apexification using MTA is provided in Figure 5.

## 4. Discussion

### 4.1. Treatment Effectiveness

Two of the retrospective studies included in this narrative review provide a comparative analysis of different methods for the treatment of permanent teeth. Kerekes et al. [50] demonstrated that apexification was more effective in younger patients aged 9–12 years, whereas conventional root canal treatment was more commonly applied in the 13–18-year-old age group. Arslan et al. [51] compared conventional endodontic treatment with revitalization and found that both methods yielded a high percentage of positive outcomes. Based on their findings, revitalization appears to be a promising option for the treatment of mature teeth with extensive periapical lesions. Nevertheless, the studies reviewed consistently reported a reduction in or the complete resolution of pain symptoms following the application of either treatment method.

### 4.2. Length of Treatment

Conventional root canal treatment is typically completed in a single visit. However, in cases involving extensive periapical lesions or intraoperative complications—such as bleeding from the canal during instrumentation—a two-visit approach may be necessary. In such instances, calcium hydroxide can be used as an intracanal medicament to reduce inflammation over a period of 1–2 weeks before final obturation. In contrast, the apexification technique involves the placement of calcium hydroxide into the root canal to induce the formation of a mineralized apical barrier. This process requires periodic monitoring and it may take between 3 to 24 months to achieve complete root-end closure.

### 4.3. Emerging and Regenerative Approaches

Researchers and clinicians are developing emerging and regenerative methods in endodontics, such as regenerative endodontic procedures (REPs) for immature teeth, platelet-rich fibrin (PRF), platelet-rich plasma (PRP), bioactive growth factors, bioceramics, and laser-assisted disinfection, which offer promising alternatives to traditional treatment. In classical revascularization techniques, a blood clot is typically used as a scaffold. However, platelet-rich plasma (PRP) concentrates, platelet-rich fibrin (PRF) gel, and platelet pellets have emerged as effective alternatives. These biologic materials offer comparable clinical and radiographic outcomes while demonstrating a reduced tendency for canal obliteration [65]. In apexification procedures, platelet-rich fibrin can be applied either in the form of a membrane or as a dense PRF gel mass used as an apical plug in teeth with open apices. Recent studies have shown that both the membrane and gel forms of PRF are equally effective as scaffolds in regenerative endodontics [66]. The success of therapy is also influenced by the effective disinfection of the root canal system. Ultrasonic irrigation is one method that enhances canal debridement. A 2010 study demonstrated that ultrasonic instrumentation combined with 0.2% chlorhexidine irrigation not only achieved effective disinfection but also accelerated the formation of the apical barrier compared to conventional hand instrumentation [67]. In more recent approaches, Er:YAG lasers used in conjunction with 0.5% sodium hypochlorite irrigation have shown promising results. This method has been associated with complete root development and the healing of periapical lesions within as little as three months [68].

### 4.4. Challenges in Dental Treatment of Permanent Teeth

Clinicians face several notable challenges when implementing emerging and regenerative treatment modalities in dentistry.

During apexification, a blunderbuss canal, defined by a large open apex with no apical stop, poses a significant obstacle to good apical sealing. Mechanical instrumentation is challenging due to the thin and weak root canal walls, which affects the obturation integrity. Using calcium hydroxide instead of MTA or Biodentine increases the risk of root fracture, makes it difficult to achieve a tight seal, and requires a longer treatment duration [69].

Revascularization also presents certain unique difficulties. A collagen matrix is required to enable the accurate implantation of mineral trioxide aggregate (MTA), and the use of a local anesthetic without vasoconstrictors is required to successfully induce bleeding. The usage of minocycline is particularly linked to the risk of tooth discoloration. Since the technique frequently requires several appointments, the most crucial prerequisite is patient compliance [70].

### 4.5. Cost-Effectiveness of Treatment

As previously noted, endodontic treatment using apexification and revitalization techniques tends to be time-consuming, requiring multiple visits and incurring higher costs. These methods also demand regular follow-up to monitor treatment outcomes. However, the studies included in this review suggest that their clinical efficacy is comparable to—and, in some cases, exceeds—that of conventional root canal treatment. CRCT, which has been widely practiced and well documented in the literature, is generally predictable and, in many cases, can be completed by an experienced clinician in a single visit. Nonetheless, research indicates that teeth become structurally weakened following root canal therapy, necessitating prosthetic reinforcement—such as a crown—to restore their strength and functionality [55]. Although regenerative endodontic procedures may involve more clinical appointments, the subsequent need for prosthetic restoration following CRCT may ultimately render it a more costly treatment modality over time.

## 5. Conclusions

This literature review indicates that regenerative dentistry has achieved notable progress compared to previous years. The currently used materials, such as ceramic silicon cements and calcium hydroxide, along with improved and more precise endodontic tools, have contributed to the enhanced effectiveness of techniques like revitalization and apexification. These advances suggest that such methods may compete with—or, in some cases, give clinical outcomes that are comparable to—conventional root canal treatment. However, given the methodological limitations and variability present in the reviewed studies (including small sample sizes, heterogeneity of protocols, and short follow-up periods), these findings should be interpreted with caution. Therefore, further well-designed, high-quality controlled trials are necessary to more definitively establish the comparative effectiveness and long-term outcomes of these regenerative approaches.

## Figures and Tables

**Table 1:** JBI critical appraisal checklist for case reports.

Study/Criteria	Was the Medical History Clearly Described?	Were the Clinical Signs Well Presented?	Were the Diagnostic Test Results Clearly Described?	Was the Treatment Adequately Described?	Was the Outcome of Treatment Clearly Presented?	Was There Justification for Publishing This Case?	Does the Event Contribute New/Significant Knowledge?	Have Ethical Issues (e.g., Patient Consent) Been Taken Into Account?
Chen, Liang, and Xiong [36], 2016	Yes	Yes	Yes	Yes	Yes	Yes	Yes	Yes
Saatchi [37], 2007	Yes	Yes	Yes	Yes	Yes	Yes	Yes	N/A
Ayna, Ayna, and Celek [38], 2009	Yes	Yes	Yes	Yes	Yes	Yes	Partially	Yes
Ahuja et al. [39], 2021	Yes	Yes	Yes	Yes	Yes	Yes	No	No
Dahiya and Singhal [40], 2022	Yes	Yes	Yes	Yes	Yes	Yes	Yes	No
Vidal et al. [41], 2016	Yes	Yes	Yes	Yes	Yes	Yes	Yes	No
Cehreli et al. [42], 2011	Yes	Yes	Yes	Yes	Yes	Yes	Yes	No

**Table 2:** JBI critical appraisal checklist for case series.

Study/Criteria	Were the Criteria for Case Inclusion Clearly Defined?	Were the Cases Representative of the Study Population?	Were Cases Appropriately Recruited?	Were Demographic and Clinical Data Clearly Described?	Were Outcomes Measured Reliably and Consistently?	Was the Length of Observation Appropriate?	Were Clinical Outcomes/ Outcome Variables Described?	Was the Data Analysis Adequate?	Was the Report Clear and Consistent with the Purpose of the Study?	Has Ethical Approval and/or Patient Consent (if Applicable) Been Described?
Paryani and Kim [43], 2013	No	Partially	N/A	Yes	Yes	Yes	Yes	Partially	Yes	No
Saoud et al. [44], 2015	No	Partially	No	Yes	Yes	Yes	Yes	Partially	Yes	No
Nageh, Ahmed, and El-Baz [45], 2018	Yes	Yes	Yes	Yes	Yes	Yes	Yes	Yes	Yes	Yes
Mejia, Donado, and Basrani [46], 2004	Yes	Yes	Partially	Yes	Yes	Yes	Yes	Partially	Yes	No
Kusgoz, Yildirim, and Gokalp [47], 2007	Yes	Yes	Partially	Yes	Yes	Yes	Yes	Partially	Yes	No
Peretz, Yakir, and Fuks [48], 1997	Yes	Yes	Partially	Yes	Yes	Yes	Yes	Yes	Yes	No
Ballikaya et al. [49], 2022	Yes	Yes	Yes	Yes	Yes	N/A	Yes	Yes	Yes	No

**Table 3:** JBI critical appraisal checklist for cohort studies.

Study/Criteria	Were the Groups Similar and Recruited from the Same Population?	Was Exposure Clearly Defined and Reliably Measured?	Was Exposure Assessed Prior to Outcome?	Were Potential Confounding Factors Identified?	Were Strategies Used to Control for Confounding Factors?	Were Outcomes Measured Fairly and Equally for All?	Was the Observation Time Sufficient?	Was the Follow-Up Complete and Adequately Described?	Were Participants Analyzed According to Group Assignment?	Was Appropriate Statistical Analysis Used?
Kerekes, Heide, and Jacobsen [50], 1980	Yes	Yes	Yes	Partially	Partially	Yes	Yes	Partially	Yes	Yes
Arslan et al. [51], 2019	Yes	Yes	Yes	Partially	Partially	Yes	Yes	Partially	Yes	Yes
Mishra et al. [52], 2025	Yes	Yes	Yes	Partially	Partially	Yes	Yes	Partially	Yes	Yes

**Table 4:** Summary table of research quality assessment (based on JBI Critical Appraisal Tools).

Author, Year	Type of Study	Yes	No	N/A or Partially	% Yes	Qualitative Assessment
Chen, Liang, and Xiong [36], 2016	Case report	8	0	0	100%	High quality
Saatchi [37], 2007	Case report	7	0	1	87.5%	High quality
Ayna, Ayna, and Celek [38], 2009	Case report	7	0	1	87.5%	High quality
Ahuja et al. [39], 2021	Case report	6	2	0	75%	Moderate quality
Dahiya and Singhal [40], 2022	Case report	7	1	0	87.5%	High quality
Vidal et al. [41], 2016	Case report	7	1	0	87.5%	High quality
Cehreli et al. [42], 2011	Case report	7	1	0	87.5%	Moderate quality
Paryani and Kim [43], 2013	Case series	5	2	3	50%	Low quality
Saoud et al. [44], 2015	Case series	5	3	2	50%	Low quality
Nageh, Ahmed, and El-Baz [45], 2018	Case series	10	0	0	100%	High quality
Mejia, Donado, and Basrani [46], 2004	Case series	7	1	2	70%	Moderate quality
Kusgoz, Yildirim, and Gokalp [47], 2007	Case series	7	1	2	70%	Moderate quality
Peretz, Yakir, and Fuks [48], 1997	Case series	8	1	1	80%	High quality
Ballikaya et al. [49], 2022	Case series	8	1	1	80%	High quality
Kerekes, Heide, and Jacobsen [50], 1980	Cohort study	7	0	3	70%	Moderate quality
Arslan et al. [51], 2019	Cohort study	7	0	3	70%	Moderate quality
Mishra et al. [52], 2025	Cohort study	7	0	3	70%	Moderate quality

**Table 5:** Research studies included in the narrative review.

No.	Author and Year	Title	Method	Teeth	Treatment Effects
Case Reports					
1	Chen, Liang, and Xiong [36], 2016	Diagnosis and Treatment of Odontogenic Cutaneous Sinus Tracts in an 11-Year-Old Boy	Conventional root canal treatment	Permanent mature molar Tooth 36—apical periodontitis	Clinical and radiological evaluation after 6 months showed that the periapical lesion had healed and the tissues on the cheek had also healed. They differed little from the surrounding area, only by a slightly discolored area on the skin.
2	Saatchi [37], 2007	Healing of large periapical lesion: A non-surgical endodontic treatment approach		Mandibular incisors—chronic periapical periodontitis	Clinical examinations after 1, 3, 5, and 12 months did not show tooth sensitivity to percussion and palpation. The radiograph showed a progressive healing process around the periapical lesions.
3	Ayna, Ayna, and Celek [38], 2009	Endodontic and prosthetic treatment of teeth with periapical lesions in a 16-year-old-girl		Nine permanent mature teeth Teeth 11–13, 21, 22, and 41–45—chronic inflammation of the periapical tissues	Clinical and radiological evaluation after 6 months showed positive treatment results. Non-surgical treatment of periapical changes in the adolescent patient brought the expected results.
4	Ahuja et al. [39], 2021	Apexification with apical growth and closure using Metapex in a necrotic immature permanent tooth with periapical abscess: A case report with 16 months follow-up.	Apexification (Metapex paste—calcium hydroxide and iodoform)	Immature permanent maxillary left central incisor tooth	Six months after treatment: the patient was asymptomatic, and radiographic evaluation showed a reduction in the periapical lesion along with notable apical development. Twelve months after treatment: the clinical condition remained stable, but the apex was still radiographically open. Sixteen months after treatment: radiographs confirmed apical closure, with continued root development and canal calcification.
5	Dahiya and Singhal [40], 2022	Apexification using Biodentine: A case report	Apexification (Biodentine)	Immature permanent tooth 21 with Ellis Class IV fracture	Two-month follow-up radiography revealed a decrease in the periapical radiolucency. The tooth remained asymptomatic, and the patient was advised to return for further monitoring. Biodentine demonstrates strong potential in the treatment of open apices, largely due to its biomimetic mineralization properties.
6	Vidal et al. [41], 2016	Apical Closure in Apexification: A Review and Case Report of Apexification Treatment of an Immature Permanent Tooth with Biodentine		Immature maxillary left central incisor after trauma	No symptoms were observed during follow-up visits at 3, 6, and 18 months. CBCT imaging at 18 months showed continuity of the periodontal ligament space, the absence of bone rarefaction, and the formation of a calcified apical barrier adjacent to the Biodentine.
7	Cehreli et al. [42], 2011	MTA apical plugs in the treatment of traumatized immature teeth with large periapical lesions	Apexification (MTA)	Two cases with immature permanent incisors Two incisors—uncomplicated fracture of the crown of the right central incisor, chronic inflammation of the periapical tissues of the upper incisors	Clinical and radiological follow-up after 18 months showed significant healing of the periapical lesions and tissue regeneration in this area.
Case Series					
1	Paryani and Kim [43], 2013	Regenerative endodontic treatment of permanent teeth after completion of root development: a report of 2 cases	Revascularization	Two cases with mature permanent incisors Case #1: Tooth 11—previously initiated, symptomatic apical periodontitis Case #2: Tooth 21—pulp necrosis, asymptomatic apical periodontitis	Case #1 (after 22 months): Asymptomatic tooth, no reaction to palpation and percussion, normal gingival pockets (<3 mm), normal reaction in the electrical test (EPT—34/80); in the X-ray image, complete disappearance of the periapical radiolucency. Treatment effect: Positive. Case #2 (after 18 months): No pulp reaction in the cold test and in the electrical test (EPT); in the X-ray image, complete lack of thinning of the bone structure, but no thickening in the apical third of the root. Treatment effect: Partially positive.
2	Saoud et al. [44], 2015	Management of Teeth with Persistent Apical Periodontitis after Root Canal Treatment Using Regenerative Endodontic Therapy		Two cases with mature permanent incisors Case #1: Tooth 21 (#9)—in the X-ray image, large space in the canal, incorrect root filling, lesion in the periapical tissues Case #2: Tooth 36 (#19)—large periapical lesion involving both the mesial and distal root areas; roots completely formed	Case #1 (after 13 months): No reaction to cold, heat, electrical test; in the X-ray image, the periapical lesion showed signs of healing. The canal space was slightly reduced due to thickening of the canal walls, and the apex seemed closed. The tooth was restored with a temporary crown. Treatment effect: Partially positive. Case #2 (after 14 months): No reaction to cold, heat, electrical test; in the X-ray image, complete healing of the periapical tissues at the distal root and slight thickening of the tissues (barrier) in the periodontal area of the mesial root. Treatment effect: Partially positive.
3	Nageh, Ahmed, and El-Baz [45], 2018	Assessment of Regaining Pulp Sensibility in Mature Necrotic Teeth Using a Modified Revascularization Technique with Platelet-Rich Fibrin: A Clinical Study		Fifteen permanent maxillary central incisors with a closed apex in patients aged 18–40 years, without gender predilection, free from systemic diseases; teeth with pulp necrosis	After 12 months: - Viable—9 teeth; - Partially viable—6 teeth. In all cases, there was no pain, swelling, or sinus tract during the follow-up period. Nine patients had hypersensitivity to the cold test. In all cases with preoperative apical radiolucency, radiographic resolution of apical periodontitis was demonstrated. After 12 months of follow-up, none of the teeth that did not show preoperative radiolucency had any bone changes.
4	Mejia, Donado, and Basrani [46], 2004	Active Nonsurgical Decompression of Large Periapical Lesions—3 Case Reports	Endo-Eze procedures Case #1 and #3: Apexification (MTA) Case #2: Conventional root canal treatment	Case #1: Maxillary central incisor (immature)—chronic apical periodontitis Case #2: Maxillary lateral incisor and canine (mature teeth)—acute suppurative apical periodontitis Case #3: Maxillary central incisor (immature)—chronic apical periodontitis	Case #1: Apexification was achieved after 14 months. Case #2: After approximately 8 months, complete healing of the periapical radiolucency was noted. Case #3: Three months after calcium hydroxide treatment, almost complete healing of the lesions was achieved.
5	Kusgoz, Yildirim, and Gokalp [47], 2007	Nonsurgical endodontic treatments in molar teeth with large periapical lesions in children: 2-year follow-up	Conventional root canal treatment	Case #1: Mandibular first molar—chronic apical periodontitis Case #2: Both mandibular first molars—chronic apical periodontitis Case #3: First and second molars—chronic apical periodontitis Case #4: Mandibular first molar—chronic apical periodontitis	In all cases, radiographs taken 3 months after treatment showed a reduction in the periapical lesions. At 2 years after treatment, the periapical lesions had healed in all cases.
6	Peretz, Yakir, and Fuks [48], 1997	Follow up after root canal treatment of young permanent molars		Twenty-eight endodontically treated first permanent molars in 18 patients aged 8 to 16 years (most approximately 12 years old) The time from completion of root canal treatment to the study ranged from 24 to 77 months.	In 16 teeth (57%), periapical changes were found before root canal treatment; in 11 teeth, the changes remained at the time of the follow-up examination (39%). No significant differences were found between the maxillary and mandibular teeth in terms of individual variables. Only 10 teeth (36%) showed complete treatment success. The overall treatment success rate in the described study was only 36% and was relatively low compared to other studies cited by the author, where it ranged between 70 and 89%.
7	Ballikaya et al. [49], 2022	The quality of root canal treatment and periapical status of permanent teeth in Turkish children and teens: a retrospective CBCT study		Total of 235 teeth of 150 patients aged 6–18 years (mean 16.0 ± 2.06 years) The study used images from cone beam computed tomography (CBCT).	Periapical lesions were found in 65.5% of root canals. The highest incidence and the largest size of periapical lesions were found in immature roots and mandibular teeth (*p* < 0.05). The main failures of endodontic treatment were as follows: - overfilling (*n* = 52); - underfilling (*n* = 93); - no filling (*n* = 46); - inhomogeneity in filling (*n* = 113) of root canals; - poor crown restoration (*n* = 85). The quality of endodontic treatment was associated with the presence of periapical lesions and their size (*p* < 0.05). Teeth with insufficiently filled, overfilled, or inhomogeneously filled root canals and poor crown restoration had a periapical lesion greater than 5 mm (*p* < 0.05). Immature teeth were most often associated with the presence of a lesion and a lesion size > 5 mm. Periapical radiolucency in young permanent teeth increased when the tooth was an incisor, had incomplete root development, or the root filling contained technical errors.
Cohort Studies					
1	Kerekes, Heide, and Jacobsen [50], 1980	Follow-up examination of endodontic treatment in traumatized juvenile incisors	Conventional root canal treatment versus apexification	Total of 166 teeth: 148 maxillary incisors and 18 mandibular incisors from patients aged 9 to 18 years (mean age 12.7 years)	Patients were recalled for clinical and radiographic examination of root fillings at 6 months and 1, 2, 3, 4, and 5 years after the end of treatment. At the end of the examination, 1 to 5 years after endodontic treatment, radiographs were reviewed jointly by the authors. The postoperative follow-up period was 1 year for 29 teeth (18%); 2 to 3 years for 92 teeth (55%); and 4 to 5 years for 45 teeth (27%). In the conventional treatment group, treatment was successful in 44 teeth (60%). In the apexification group, treatment was successful in 88 teeth (95%). In the conventional treatment group, 62 root canals (85%) were adequately sealed, and optimal root filling was achieved in 65 teeth (90%). Excess root filling material greater than 1 mm was found in eight cases (11%). In the apexification group, 90 root canals (97%) were adequately sealed, and the optimal level of root filling was achieved in 91 teeth (98%). Excess root filling material greater than 1 mm was observed in two cases (2%) The technical level of root fillings and treatment outcomes were significantly higher and more effective in the apexification group (95%) than in the conventional treatment group (60%). The strongest influence on treatment success was the quality of root filling sealing and the method used in the treatment groups (35%). The results showed that the original, standardized technique was the preferred method of treatment for patients aged 13 to 18 years. However, in the treatment of patients aged 9–12 years, apexification and modified obturation techniques proved more effective.
2	Arslan et al. [51], 2019	Regenerative Endodontic Procedures in Necrotic Mature Teeth with Periapical Radiolucencies: A Preliminary Randomized Clinical Study	Revascularization (group #1) versus conventional root canal treatment (group #2)	Total of 56 permanent teeth in 49 patients Group #1: Teeth treated with regenerative endodontic procedures (REP): 28 teeth Group #2: Teeth treated with conventional endodontic treatment (CRCT): 28 teeth	Comparison of the results of treatment with regenerative endodontic procedures (REP) with those of conventional root canal treatment (CRCT) in mature permanent teeth. With a follow-up rate of approximately 73.4% across all patients for 12 months, favorable clinical and radiographic results were observed in 92.3% for REP and 80% for CRCT, respectively, and the difference was not statistically significant. Half of the teeth treated with REP responded to the electric pulp test.
3	Mishra et al. [52], 2025	Apexification of the mineral trioxide aggregate in nonvital immature anterior teeth with and without platelet-rich plasma: A preliminary clinical study	Group #1: Apexification (MTA without PRP) Group #2: Apexification (MTA with PRP)	Group #1: 18 immature permanent anterior maxilla non-vital teeth Group #2: 18 immature permanent anterior maxilla non-vital teeth	The clinical and radiographic outcomes were evaluated at 6 and 12 months. Periapical healing was observed in both groups, with excellent healing seen in 11 roots in group #1 and 17 roots in group #2. Apical closure was achieved in 100% of group #2 and 77% of group #1. These findings suggest that single-visit apexification using MTA combined with platelet-rich plasma (PRP) is a conservative, time-efficient, and effective approach for managing non-vital immature teeth with open apices.

## Data Availability

All raw data are available from the corresponding author on reasonable request.

## Supplementary Materials

The following supporting information can be downloaded at https://www.mdpi.com/article/10.3390/jcm14145083/s1. Table S1: Search strategy.

## Abbreviations

The following abbreviations are used in this manuscript:

AAE	American Association of Endodontists
Ca(OH)_2_	Calcium hydroxide
CRCT	Conventional root canal treatment
DAP	Double-antibiotic paste
EDTA	Ethylenediaminetetraacetic acid
Er:YAG	Erbium-doped yttrium aluminum garnet
ESE	European Society of Endodontology
MeSH	Medical Subject Headings
MTA	Mineral trioxide aggregate
NaOCl	Sodium hypochlorite
PRF	Platelet-rich fibrin
PRP	Platelet-rich plasma
REP	Regenerative endodontic procedures
SAP	Single-antibiotic paste
SCAP	Stem cells from the apical papilla
TAP	Triple-antibiotic paste
