# Supplementary material for: Effectiveness of Treatment of Periapical Lesions in Mature and Immature Permanent Teeth Depending on the Treatment Method Used: A Critical Narrative Review Guided by Systematic Principles
Source: J Clin Med. 2025 Jul 17;14(14):5083. doi: 10.3390/jcm14145083 (PMC12295325; doi:10.3390/jcm14145083)
Supplement: Supplementary file 1 [file jcm-14-05083-s001.zip › jcm-3728122-supplementary.pdf]

Supplementary material.

Table S1. Search strategy.

Order	Term
**#1**	“Apexification”
**#2**	“Revascularization”
**#3**	“Conventional Root Canal Treatment”
**#4**	“Immature Permanent Teeth”
**#5**	“Permanent Teeth”
**#6**	“Periapical Lesions”
**#7**	#1 OR #2 OR #3
**#8**	#4 OR #5
**#9**	#6
**#10**	#7 AND #8 AND #9
